# Development of nanoparticle-based Toll-like receptor agonists for respiratory immunotherapy

**DOI:** 10.17179/excli2025-9020

**Published:** 2026-01-07

**Authors:** Amisha S. Raikar, Ananya Verma, Mayuri B. Naik, Sweta M. Prabhu, Keshav Raj Paudel, Kamal Dua

**Affiliations:** 1 Department of Pharmaceutics- PES Rajaram and Tarabai Bandekar College of Pharmacy, Ponda, Goa, 403401, India; 2 Department of Biomedical, Nutrition and Sports Sciences, Newcastle University, Newcastle upon Tyne, NE1 7RU, United Kingdom; 3 Department of Biotechnology- Amity Institute of Biotechnology, Amity University, Mumbai-Pune, Maharashtra, 410206, India; 4 Department of Pharmacology- PES Rajaram and Tarabai Bandekar College of Pharmacy, Ponda, Goa, 403401, India; 5 Department of Pharmaceutics, Srinivas College of Pharmacy, Mangalore, Karnataka, 574143, India; 6 NICM Health Research Institute and School of Science, Western Sydney University, Westmead, NSW, 2145, Australia; 7 Woolcock Institute of Medical Research, Macquarie University, Sydney, New South Wales, Australia

**Keywords:** respiratory diseases, Toll-like receptors (TLRs), nanoparticle-based immunotherapy

## Abstract

Respiratory diseases are global health challenges demanding innovative immunotherapy solutions. Toll-like receptors (TLRs) play a crucial role in immune responses, making them attractive therapeutic targets. This review examines nanoparticle-based Toll-like receptor agonists as a promising approach for respiratory immunotherapy. Nanoparticles offer targeted drug delivery and sustained release, ideal for enhancing TLR agonist efficacy. This article explores TLRs' role in immunomodulation, nanoparticle applications, design considerations, preclinical efficacy, safety assessments, challenges, and future prospects. Promising results from animal studies suggest enhanced immunological responses and reduced inflammation compared to conventional treatments. Safety concerns are addressed with insights from toxicity studies. Challenges include regulatory hurdles and biocompatibility, with strategies proposed for optimization. Nanoparticle-based TLR agonists hold great potential to transform respiratory disease treatment, warranting further research and collaboration for successful clinical translation.

See also the graphical abstract(Fig. 1).

## Highlights

- Nanoparticles provide an efficient platform for targeted delivery of Toll-Like Receptor (TLR) agonists to the respiratory system.
- TLR agonists, whether synthetic or natural, demonstrate significant potential in modulating immune responses within respiratory tissues.
- Nanoparticle-based TLR agonists amplify immune responses within the respiratory tract, including the activation of immune cells and cytokine production.
- Strategies for precise and localized delivery minimize off-target effects, optimizing therapeutic outcomes.
- Addressing safety concerns associated with nanoparticle-based therapies, including toxicity mitigation strategies.
- Insights from preclinical and clinical studies underscore the efficacy and potential of nanoparticle-based TLR agonists in respiratory disease treatment.

## 1. Introduction

Respiratory diseases, which are the chief contributor of morbidity and mortality, place a huge strain on worldwide healthcare systems (James et al., 2017[28]). They include a broad range of health conditions, including asthma, chronic obstructive pulmonary disease (COPD), and various respiratory infections, the severity of which is aggravated by harmful environmental, occupational, and behavioural exposures. In addition to avoiding allergens and using drugs to manage symptoms, allergen immunotherapy, also known as immunologic desensitisation or hypo sensitization treatment administration, has proven to be beneficial in treating IgE-mediated respiratory illnesses such as allergic rhinitis, comprising rhinorrhoea, nasal obstruction, nasal itching, and sneezing (Ciprandi et al., 2015[12]) and asthma. The two most prescribed methods for this therapeutic intervention are sublingual immunotherapy (SLIT) and subcutaneous immunotherapy (SCIT) (Muraro et al., 2018[49]). Although it has been more than a century since Noon and Freeman described immunotherapy (Cohen et al., 2003[13]), as humanity evolves, research and development of advanced treatment options for immunotherapy to augment patient well-being and quality of life, especially in regions where access to healthcare and resources for treatment are limited, have become the need of the hour. One of the most promising techniques to defend against allergic respiratory disorders is the application of toll-like receptors (TLRs) (Casale and Stokes, 2011[11]). The TLR family was discovered when Toll, an insect receptor that establishes dorsoventral polarity during embryogenesis, was identified (Ljashimoto et al., 1988[42]). Subsequent research demonstrated that Toll plays a crucial function in the innate immune response of insects against fungal infection (Lemaitre et al., 1996[40]). TLR was the first protein family to exhibit the characteristics postulated by Janeway for pattern recognition receptors (PRRs) (Janeway, 1989[29]). Pattern recognition receptors (PRRs) function as proteins that are encoded in the germline and have the ability to identify conserved microbial products, commonly referred to as "Pathogen-Associated Molecular Patterns" (PAMPs), as well as molecules that are produced by damaged cells, known as "Damage-Associated Molecular Patterns" (DAMPs). Upon activation, TLRs initiate a series of immune reactions, which involve the generation of pro-inflammatory cytokines and the mobilization of immune cells. This recognition and response by TLRs are vital for the body's defence against infections and for maintaining immune homeostasis. Immunoadjuvant TLR agonists provide resistance against microbes through epigenetic modifications and metabolic reprogramming, thus enhancing an individual's immunity (Owen et al., 2021[52]).

The staggering scope of nanoparticles cannot be emphasised enough; their nanoscale adaptability, multidimensionality, and absorption properties account for their versatility (Khan et al., 2019[33]). The alteration within their building blocks results in variations at both the physiochemical and molecular levels, thereby enhancing their value in the realm of biomedicine, not just in drug delivery but also as immunotherapeutic systems (Shetab et al., 2018[68]). The transmutational abilities of nanoparticles facilitate their pathogen mimicry and employment in the immune system. The prospect of combining nanoparticles with immunity stimulant TLR agonists is conducive to combating infectious respiratory disorders (Knuschke et al., 2014[36]).

Agonists targeting TLRs for allergy treatment are in various stages of development and have been tested in pre-clinical models, but only some have reached clinical evaluation (Kirtland et al., 2020[35]). The objective of this review is to elaborate on the scope, development, and immunomodulatory effects of nanoparticle-based TLR agonists in relation to respiratory diseases.

## 2. Toll-Like Receptors and Immunotherapy

The germline-encoded transmembrane proteins known as TLRs, which are derived from the toll gene family, are essential for detecting numerous microbial patterns and triggering the innate immune system. Distinct types of white blood cells have distinct TLR expressions. They are found on subsets like natural killer (NK) cells, T and B lymphocytes, dendritic cells (DCs), macrophages, and non-immune cells such fibroblasts, endothelial cells, and epithelial cells (Pahlavanneshan et al., 2021[53]) 

TLR4 can be found in both the cell's outer membrane and within its internal compartments. Among the extracellular TLRs (such as TLR1, TLR2, TLR4, TLR5, TLR6, and TLR10), they are situated on the outer cell membrane, while the intracellular TLRs (comprising TLR3, TLR7, TLR8, and TLR9) are positioned within endosomes and the endoplasmic reticulum. These molecules have the ability to recognize infectious agents through their leucine-rich repeat (LRR) domain, which is situated on the cell's outer surface or within luminal compartments. They then transmit signals via their conserved cytosolic toll-like/interleukin-1 receptor (TIR) homology domain to activate downstream signaling adaptor proteins like myeloid differentiation primary response gene 88 (MyD88) (Hoden et al., 2022[23])

The innate immune response identifies invading pathogens within the host through receptors called pattern recognition receptors (PRRs). This group includes Toll-like receptors (TLRs), NOD-like receptors (NLRs), RIG-I-like receptors (RLRs), and cytosolic DNA sensors. TLRs are capable of recognizing both pathogen-associated molecular patterns (PAMPs) and endogenous damage-associated molecular patterns (DAMPs). When TLRs transmit signals, it leads to the production and release of pro-inflammatory cytokines and co-stimulatory molecules (Vidya et al., 2018[80]). These molecules play a crucial role in supporting inflammatory responses and attracting neutrophils and macrophages to the sites of inflammation.

In both the innate and adaptive immune systems, TLRs play a crucial role. Their ability to detect both external PAMPs and internal DAMPs enables them to initiate ligand-mediated signal transduction, ultimately leading to the initiation of an inflammatory response. Recent evidence has been increasingly supportive of the significance of TLRs and their ligands in various pathological conditions, including inflammation, cancer, and autoimmune disorders (Engel et al., 2011[19]). TLRs also play a substantial role in the fields of vaccination and immunotherapy. The bioavailability, half-life, clearance, and toxicity of TLR agonists are pivotal factors influencing the enhancement of therapeutic effectiveness.

Immune system activation-TLRs identify conserved pathogen-associated molecular patterns and structural subunits, such as virulence proteins (such as flagellin) and microbial cell wall (such as peptidoglycan) and cell membrane (such as LPS), which are not present in the host (Barton and Medzhitov, 2003[10]). The innate immune system's surveillance of pathogens with these conserved structures is made possible by TLRs' recognition of pathogen-associated molecular sequences. TLR activation leads to the development of dendritic cells and inflammatory responses, which eliminate invasive infections. TLRs serve as a crucial bridge between the innate and adaptive immune systems, a fact underscored by their role in facilitating the maturation of dendritic cells, which is essential for generating pathogen-specific adaptive immune responses. To illustrate, TLR3 recognizes double-stranded RNA, commonly generated during viral infections, while TLR4 identifies the lipopolysaccharide found in Gram-negative bacteria. One of the key features of this microbial recognition system is that TLRs initiate signaling pathways vital for triggering an immune response tailored to the specific pathogenic challenge. TLRs effectively connect the activation of antigen-presenting cells, specialized cells responsible for kickstarting T lymphocyte activation and initiating adaptive immunity, with the identification of microbial invaders (Riccioli et al., 2006[60]).

### 2.1 Toll-like receptor (TLR) signaling pathways

**Figure 2:**
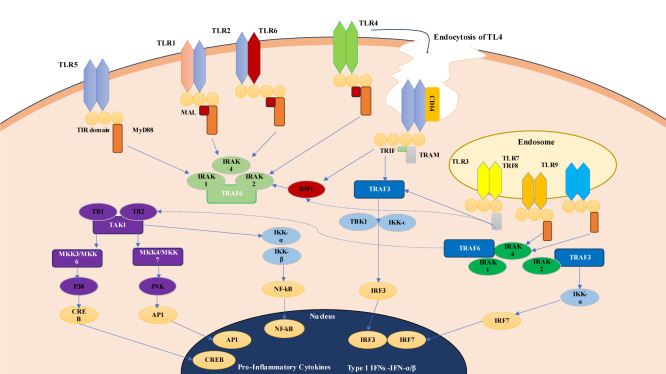
Figure 2 presents an encompassing view of the signaling pathways associated with Toll-like receptors (TLRs). This schematic provides insights into the localization of TLRs, the key signaling molecules involved, and the downstream pathways they activate. Notable components include activator proteins (AP), cAMP response element binding protein (CREB), interferons (IFN), interleukin-1 receptor-associated kinase (IRAK), IFN regulatory factors (IRF), IkB kinase α (IKKα), c-Jun N-terminal kinase (JNK), mitogen-associated protein kinase kinase (MKK), nuclear factor-κB (NF-κB), receptor-interacting protein (RIP), TANK-binding kinase 1 (TBK), Toll-interleukin-1 receptor (TIR), tumour necrosis factor receptor-associated factor (TRAF), TRIF-related adaptor molecule (TRAM), and TIR-domain containing adaptor-inducing IFN-β (TRIF). This comprehensive visual aids in understanding the intricate TLR signaling network.

Toll-like receptors (TLRs) represent a fundamental component of the innate immune system with diverse roles in detecting and responding to microbial infections and tissue damage. They are strategically localized on the cell surface or within intracellular compartments like endosomes, allowing them to sense a wide range of pathogens as shown in Figure 2(Fig. 2). TLRs recognize specific molecular signatures associated with microbes, such as lipopolysaccharides (LPS) from bacteria (TLR4) or double-stranded RNA from viruses (TLR3). These receptors transmit signals via adapter proteins like MyD88 (utilized by most TLRs except TLR3) and TRIF (used by TLR3 and some others), initiating intricate signaling cascades. Once activated, TLR signaling converges on nuclear factor-kappa B (NF-κB) and interferon regulatory factors (IRFs), transcription factors that migrate to the nucleus and orchestrate the expression of various immune genes (Kawasaki and Kawai, 2014[32]). This results in the secretion of pro-inflammatory cytokines, chemokines, type I interferons, and other immune effectors. These molecules collectively shape the immune response by recruiting immune cells to sites of infection, enhancing antigen presentation by antigen-presenting cells (APCs), and establishing an antiviral state in neighbouring cells (O'Neill, 2006[51]). Beyond their immediate role in innate immunity, TLRs serve as crucial mediators connecting the innate and adaptive immune systems. Upon activation, TLRs stimulate APCs, particularly dendritic cells, to process and present pathogen-derived antigens to T lymphocytes (Barton and Medzhitov, 2003[10]). This interaction is pivotal for the initiation of adaptive immune responses, including the activation of T cells and B cells. Consequently, TLRs have profound implications not only in the defence against infections but also in vaccine development, where they are harnessed to enhance the efficacy of vaccines, and in immunotherapies, where they play a role in modulating immune responses against diseases like cancer.

### 2.2 TLR-mediated epigenetic and metabolic reprogramming

Activation of TLRs can lead to modifications in histones, DNA methylation patterns and non-coding RNAs as well. For example stimulating TLR4 in macrophages has been shown to increase histone acetylation at promoters of genes encoding pro-inflammatory cytokines such as TNF-α and IL-6 which leads to boosting of their expression) (Hoden et al., 2022[23]). Similarly, TLR7 and TLR8 activation can alter microRNA levels, like miR-146a, which act to fine-tune the immune response and prevent excessive inflammation) (Hoden et al., 2022[23]). These changes can persist beyond the initial stimulus which gives rise to a type of innate immune memory, sometimes called trained immunity, which allows immune cells to respond more efficiently to subsequent infections.

TLR activation also reshapes cellular metabolism. Activated macrophages and dendritic cells often switch from relying primarily on oxidative phosphorylation to aerobic glycolysis, which provides rapid energy and the building blocks needed for cell growth and cytokine production. Changes in metabolism also affect mitochondrial activity, fatty acid processing, and the production of reactive oxygen species, all of which feed back into immune signaling and even influence epigenetic modifications. Metabolism and epigenetics are closely linked. Molecules like acetyl-CoA and S-adenosylmethionine, which are generated during metabolic processes, are essential for histone acetylation and DNA methylation. This means that the metabolic state of a cell can directly impact gene regulation while epigenetic changes can in turn affect genes that control metabolism. 

## 3. Nanoparticles in Immunotherapy

Nanoparticles (NPs) present a promising avenue for delivering immunomodulatory agents to immune cells, effectively regulating their responses. Several investigations have demonstrated that nanoparticles (NPs), primarily because of their size, engage with both innate and adaptive immune cells in a manner reminiscent of interactions with pathogens such as bacteria or viruses. These interactions prompt immune cells to perceive NPs either as entirely foreign entities or by establishing precise interactions with individual NP components. Consequently, these engagements trigger immune responses that can either activate or suppress immune cells, with the specificity of the response depending on the properties of the NPs. These interactions hold potential for therapeutic applications. Progress in nanotechnology empowers the intentional customization of nanoparticles (NPs) by integrating targeting and pharmacological components into their nanostructures. This pioneering strategy facilitates novel means of interaction with the immune system, presenting opportunities for the treatment of diverse diseases. Through precise engineering, NPs can possess specific compositions and physicochemical attributes that modulate their interactions with immune cells, thus affecting recognition, uptake, and distribution within organs. Strategic molecular design allows NP surfaces to carry decorations that target specific receptors, as well as controlled release of encapsulated payloads at desired sites.

NPs serve as versatile carriers for various immunotherapeutic. Examples include NPs loaded with cytokines, peptides, adjuvants, or specific nucleic acids, tailored for efficient delivery to affected tissues or cells (Anfray et al., 2020[5]). NPs can also be designed as vaccine formulations against conditions such as cancer, autoimmune disorders, or infectious diseases, bolstering the immune system's disease-fighting capabilities. Innovative immune checkpoint inhibitors and monoclonal antibodies can be enclosed within NPs to enhance targeted delivery and minimize systemic side effects (Anfray et al., 2020[5]). NPs can be engineered to transport small-molecule immunomodulatory drugs to specific intracellular compartments within particular immune cell populations. 

Nanoparticles can be engineered to deliver therapeutic agents specifically to target cells or tissues, mitigating unintended side effects and enhancing the overall effectiveness of the treatment. One such example is Precise Transport of Therapeutic Nucleic Acid for Enhancing Cancer Immunotherapy (Lächelt and Wagner, 2015[38]). The emergence of intelligent nanocarriers for utilizing nucleic acids in cancer immunotherapy presents a significant avenue, especially in terms of safeguarding these genetic materials from degradation while enhancing precise delivery. Unlike alternative delivery techniques like electroporation, gene guns, and sonoporation, nanoparticles stand out due to their versatility and distinctive magnetic, optical, chemical, and structural attributes, which confer a range of benefits for overcoming biological hurdles. These advantages encompass improved precision in delivering nucleic acids to specific targets, effective intracellular processing and movement, favorable biodistribution and pharmacokinetic characteristics, and a regulated release mechanism facilitated by skillful nanoparticle engineering.

In study conducted by Liu et al., 2022, showed that Disulfiram (DSF), originally used for alcoholism, shows anticancer potential when combined with copper ions (Cu2+) (Liu et al., 2022[41]). DSF's low water solubility and the toxicity caused by external Cu2+ limit its application. To address this, pH-responsive lipid-coated calcium phosphate nanoparticles (LCP NPs) were designed to carry both Cu2+ and DSF. These nanoparticles, injected intravenously, accumulate in tumors due to their prolonged blood half-life. Inside the acidic tumor environment, the nanoparticles break down, releasing Cu2+ and DSF. This results in the formation of a cytotoxic metabolite, bis(diethyldithiocarbamate)-copper (CuET), which induces cancer cell death. CuET not only directly kills cells but also triggers immunogenic cell death (ICD), reshaping the immunosuppressive tumor microenvironment. This enhances the effectiveness of immune checkpoint blockade (ICB) therapy, promoting systemic immune responses.

Nanoparticles can evade the body's immune system and prolong their presence in circulation, allowing sustained release of therapeutic agents and reducing the need for frequent dosing. Researchers Zhang et al. (2023[87]) developed a magnetic nanoparticle delivery system that simultaneously carries indocyanine green (ICG) for PTT and the immunostimulatory R837 hydrochloride (R837) (Qin et al., 2023[58]; Zhu et al., 2023[88]). This system consists of a core made of Fe3O4 magnetic nanoparticles (MPs) loaded with ICG, coated with polyethylene glycol polyphenols (DPA-PEG) to hold R837. Upon intravenous injection, the MIRDs demonstrate prolonged circulation, enable magnetic resonance imaging (MRI) guidance, and allow magnetic targeting.

Nanoparticles can shield therapeutic agents from degradation and enzymatic breakdown, ensuring that a higher concentration reaches the target site. Multiple therapeutic agents or modalities can be loaded onto nanoparticles, allowing for combination therapy and synergistic effects. Nanoparticles can also be tailored for individual patients, considering factors such as genetic makeup and disease characteristics.

## 4. Designing Nanoparticles for Respiratory System Targeting

**Figure 3:**
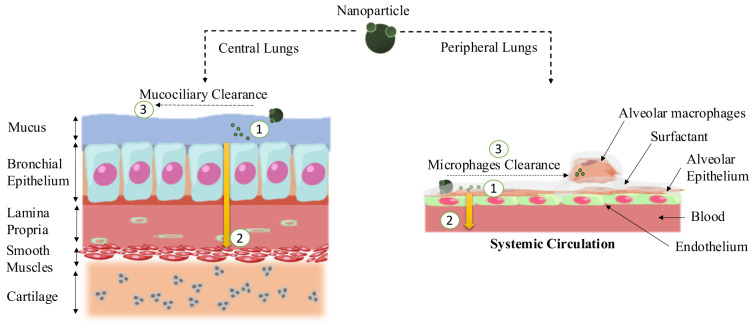
Barriers that drugs or nanoparticles face upon encountering the lung environment. Once the nanoparticles come in contact with the lung lining fluids (1), they need to traverse the pulmonary epithelium (2) to access the underlying tissue or enter the systemic circulation. These entities must overcome efficient clearance mechanisms (3), which involve mucociliary clearance within the airways and clearance by macrophages in the deeper lung regions.

In comparison to systemic methods like oral or intravenous administration, using the pulmonary route (through intratracheal or intranasal pathways) enables nanoparticles to reach the lungs directly. This approach bypasses initial metabolism, leading to higher concentrations of therapeutic agents within lung tissues. Due to variations in airway diameters across different lung regions, the distribution of inhaled nanoparticles becomes uneven. Hence, the design of nanoparticles needs to match the respiratory tract's structure to achieve optimal delivery. Figure 3(Fig. 3) illustrates the barriers that drugs or nanoparticles face during pulmonary drug delivery.

### 4.1 Particle size

Numerous research investigations have delved into the impact of nanoparticle size on their uptake within the respiratory system. Larger particles, typically with diameters surpassing 5 μm, exhibit a propensity to become trapped within the upper airways, whereas smaller particles within the range of 1-5 μm are better suited for deposition in the lower airways. Particles below the 500 nm size threshold can reach the alveoli via Brownian diffusion. Importantly, nanoparticles measuring less than 200 nm have exhibited a remarkable ability to effectively target respiratory epithelial cells, thus evading clearance by macrophages (Sung et al., 2007[74]).

### 4.2 Overcoming the mucosal barrier

Aerosol-based nanoparticle delivery to the lungs, aimed at precise treatment with reduced systemic toxicity, holds immense potential. A significant challenge arises from the airway's protective mucus layer, produced by airway goblet cells (Suk et al., 2014[73]).

When inhaled, nanoparticles encounter the swift mucociliary clearance process in the respiratory system, offering a limited timeframe for effective lung delivery. To address this, nanoparticles must first breach the outer mucus layer and then rapidly navigate the adherent internal layer. Larger particles and those interacting strongly with mucus are quickly cleared through mucociliary mechanisms. This underscores the importance of designing smaller nanoparticles, with recent evidence indicating that particles below 200 nm exhibit enhanced mucus penetration. Nanoparticles' ability to traverse the mucus barrier correlates with their size, favouring smaller counterparts (Mastorakos et al., 2015[45]). To minimize mucus interaction, nanoparticles with hydrophilic attributes and neutral surfaces, often incorporating materials like PEG, are preferred. A novel approach involves a brush-like triblock polymer coating comprising PEG and polypropylene glycol, were coating thickness and density impact interactions with mucus and lung surfactant (Tang et al., 2009[75]). Alongside PEG, alternative methods are emerging, including the use of mucus-penetrating peptides as nanoparticle coatings to improve mucus penetration and cellular uptake. Another strategy involves incorporating mucolytics into nanoparticle design. Agents that disrupt disulfide bonds, such as N-acetyl-cysteine, and mucolytic enzymes like papain and bromelain, show promise in nanoparticle formulations (Witten et al., 2018[86]). A recent innovation includes a "nano-into-micro" dry powder developed for ivacaftor delivery (Porsio et al., 2020[56]). This formulation integrates mannitol and cysteamine to alleviate viscosity and enhance mucus diffusion.

### 4.3 Evasion of alveolar macrophage clearance for nanoparticles

Before achieving targeted delivery to epithelial cells through the pulmonary route, addressing the crucial concern of nanoparticle clearance by airway and alveolar macrophages is important. Key factors influencing macrophage clearance encompass nanoparticle size, surface charge, and surface characteristics. Phagocytosis is the primary mode of clearance for nanoparticles spanning the range of 500 nm to 6 μm (Qie et al., 2016[57]). Consequently, meticulous nanoparticle design must consider size as a pivotal parameter. Surface charge, or potential, is equally significant to evade alveolar macrophage interactions. Positively charged (cationic) nanoparticles have a tendency to be more readily engulfed by alveolar macrophages, whereas negatively charged (anionic) nanoparticles are better at evading macrophage clearance. Importantly, cationic nanoparticles can trigger internalization by pulmonary dendritic cells, leading to their recruitment and maturation, whereas anionic nanoparticles typically do not elicit an immune response. Various polymers, such as PEG, polyvinyl alcohol, and zwitterionic polymers, have shown promise in reducing macrophage interaction when applied to nanoparticle surfaces. For instance, after PEG coating, there was a notable decrease in particle uptake by alveolar macrophages (Shen et al., 2015[67]). In nanoparticle synthesis, two primary strategies are employed to overcome both the mucus barrier and nonspecific macrophage uptake. The first strategy involves carriers like polyplexes, mesoporous nanoparticles, and liposomes, used to transport therapeutic agents to respiratory epithelial cells. The second strategy utilizes mucolytic agents to facilitate the penetration of nanoparticle carriers through the mucus barrier, ultimately targeting the airway epithelium upon pulmonary administration.

### 4.4 Enhancing nanoparticle uptake by the epithelial membrane

Once the obstacles of mucus and macrophage clearance have been overcome, nanoparticles reach the surfaces of epithelial cells. At this stage, the focus shifts to epithelial endocytosis, a crucial element in nanoparticle design. Typically, nanoparticles are engineered with specific ligands that engage with epithelial receptors or adhesion molecules. For instance, nanoparticles conjugated with Vitamin B12 exhibit increased cellular uptake by respiratory epithelial cells, owing to the presence of Vitamin B12 receptors on these cell surfaces (Fowler et al., 2013[20]). Likewise, cell-penetrating peptides offer an alternative approach to enhance nanoparticle uptake by respiratory epithelium. These peptides act as amphiphilic carriers for delivering proteins to airway epithelial cells. Innovations also extend to designing glycosaminoglycan-binding enhanced transduction peptides to augment cellular uptake (Abu-Awwad et al., 2017[1]). Nanoparticles chemical composition can be tailored for optimal epithelial cell uptake. Comparative studies have showcased varied uptake abilities among nanoparticles, highlighting the importance of composition. The non-invasive aerosol inhalation method has gained attention for epithelial targeting, with stabilized mRNAs delivered using hyperbranched poly-β-amino ester nanoparticles (PBAEs) (Wang et al., 2022[82]). Through nebulized delivery, these PBAE nanoparticles achieved substantial targeting of lung epithelial cells, offering promising clinical delivery systems. Intravenous injection can also be employed, provided nanoparticles feature specific modifications for heightened epithelial cell uptake. An example involves the creation of liposome-based nanoparticles that integrate nanobodies tailored for surfactant-associated protein (Balmert and Little, 2012[8]). These nanobody-linked liposomes efficiently transported therapeutic agents into precise lung epithelial cells, demonstrating promise in various disease scenarios.

### 4.5 Respiratory tract particle clearance dynamics

Inhaled particles face diverse clearance pathways contingent on their attributes and regional distribution. Primarily, three mechanisms govern this process: muco-ciliary clearance, phagocytosis, and systemic uptake. Muco-ciliary clearance takes precedence in upper airways, orchestrated by the ciliated columnar epithelium. This epithelium produces mucus, capturing particles and setting them in motion through ciliary beats, eventually leading to expulsion through coughing or swallowing. Particles exceeding 5 μm in size are predominantly eliminated within the upper airways through this mechanism. In contrast, smaller particles settled deeper in the lungs experience reduced muco-ciliary action, leading to extended retention. Phagocytosis plays a diminished role in upper airways, with macrophages present but less dominant. Deep lungs entail more intricate clearance mechanisms determined by particle dissolution kinetics. Slower-dissolving or insoluble particles might interact with epithelial and immune cells, undergoing removal through muco-ciliary clearance, phagocytosis by alveolar macrophages, or endocytosis. Alveolar macrophages take the lead in deep lung clearance, internalizing particles, then either digesting them or exiting into the lymph or through muco-ciliary clearance. Phagocytosis chiefly accounts for particle clearance in the 1-5 μm range. Sub-200 nm particles often escape macrophage recognition, potentially due to size or rapid uptake by epithelial cells (Stuart, 1984[71]). A portion of nanoparticles can migrate into the systemic bloodstream via protein/receptor-mediated uptake, and some might enter systemic circulation through endocytosis via alveolar caveolae. For nanoparticles rapidly dissolving in the deep lungs, drug release occurs promptly, leading to systemic circulation absorption. The rate of absorption depends on drug characteristics, particularly lipophilicity and molecular weight. Low-molecular-weight lipophilic drugs tend to be absorbed most rapidly.

### 4.6 Safety and toxicity issues

A significant concern surrounding nanoparticle therapeutics revolves around potential unforeseen adverse health impacts. Experiments conducted on rodents have suggested that when carbon nanoparticles are administered intratracheally, they can expedite vascular thrombosis (Medina et al., 2007[46]). Similarly, inhaled iridium particles have shown the potential to move from the lungs into the systemic circulation, giving rise to concerns regarding possible adverse vascular effects (Semmler et al., 2004[65]). Inhaled carbon nanoparticles have been observed to reach the brain, although the extent of their impact on the central nervous system remains uncertain. Intriguingly, recent research suggests that the cytotoxicity of metallic nanoparticles is comparable to that of metallic microparticles, implying that size might not be the primary determinant of toxicity. Nanoparticle toxicity remains a serious issue and necessitates thorough investigation. Beyond inherent nanoparticle toxicity, the materials employed for nanoparticle formulation could also shows toxic effects, potentially limiting their viability for therapeutic products. As an example, the harmful effects of polycyanoacrylates have been evidenced, with specific forms leading to an elevation in lactate dehydrogenase (LDH) activity within human pulmonary epithelial cells (Stone et al., 2006[70]). The toxicity level was influenced by the type of polycyanoacrylate, where shorter chain variants exhibited greater cytotoxicity. Similarly, polyethyleneimine (PEI) has displayed cytotoxicity in lung cells. Interestingly, studies indicate that particle toxicity in the lungs might hinge more on material selection than on particle size. For instance, comparisons between PLGA nanoparticles and polystyrene particles of comparable size suggest that the choice of material plays a crucial role in determining lung nanoparticle toxicity (Dailey et al., 2006[15]).

### 4.7 Epigenetic effects of nanoparticles

Nanoparticles are increasingly recognized not just for their ability to deliver drugs but also for their influence on epigenetic regulation in immune cells. By affecting DNA methylation, histone modifications and even non-coding RNA expression nanoparticles can change the expression of genes involved in inflammation and Toll-like receptor (TLR) signaling. For example, gold nanoparticles have been reported to reduce DNA methylation at promoters of pro-inflammatory genes in macrophages which can enhance cytokine production and support immune activation (Wang et al., 2022[82]). Also chitosan based nanoparticles have been shown to increase histone acetylation leading to higher expression of TLR4 and stronger downstream NF-κB signaling (Kumar et al., 2003[37]). PLGA nanoparticles loaded with TLR agonists can influence the expression of microRNAs such as miR-155 and miR-146a in dendritic cells, helping to balance pro- and anti-inflammatory signals and modulate adaptive immune responses (Dailey et al., 2006[15]).

In respiratory immunotherapy these epigenetic effects are particularly important. Nanoparticles can be designed to selectively influence TLR pathways in airway epithelial cells and alveolar macrophages which than enhances their protective immune responses while minimizing harmful inflammation. Understanding these epigenetic interactions can guide the design of nanoparticles with optimal size, composition, and surface chemistry to achieve targeted and safe immunomodulation. 

## 5. Recent Developments in Nanoparticle-Based Drug Delivery for Respiratory Diseases

Recent progress in the realm of nanoparticle for drug delivery for respiratory diseases has ushered in exciting advancements. These innovations hold the promise of revolutionising treatment approaches for various pulmonary conditions. Through careful design and engineering, nanoparticles have emerged as versatile carriers capable of navigating the intricate barriers within the respiratory system. By capitalizing on their size, surface properties, and targeting ligands, researchers have managed to improve the precision and effectiveness of drug delivery to the pulmonary system. This has the potential to improve treatment outcomes, minimize systemic side effects, and enable the targeted transport of therapeutic substances to the areas of concern. 

### 5.1 COVID-19 treatments

Nanoparticles have been explored for delivering antiviral drugs and vaccines directly to the respiratory system to combat the SARS-CoV-2 pandemic. Several companies are vigorously pursuing the development of mRNA vaccines encoding viral proteins like the spike protein, encapsulated within nanoliposomes possessing physicochemical properties that resonate with immunization strategies aimed at specific tumour antigens (Dheyab et al., 2021[17]). Crafting such nanocarriers that can elude scavenger cell recognition while maintaining nontoxic and nonimmunogenic profiles presents a formidable challenge, necessitating substantial time before clinical availability. Moderna achieved a significant milestone on March 16, 2020, by commencing a Phase I clinical trial for an mRNA vaccine known as mRNA-1273, which was enclosed within lipid nanoparticles, a mere 63 days after the selection of the genetic sequence (Hodgson, 2020[24]). Collaborating with the Vaccine Research Center at the U.S. National Institutes of Health, the trial successfully enrolled its initial participants, marking swift progress. Similar initiatives are in progress by CureVac and BioNTech, with the latter in collaboration with Pfizer. The Pfizer/BioNTech partnership has embarked on Phase I/II trials for their vaccine candidates (Dheyab et al., 2021[17]). Inovio Pharmaceuticals' exploration of DNA plasmid vaccines yielded promising results in animal studies, prompting the initiation of Phase I human trials (Dheyab et al., 2021[17]).

Another Phase I candidate, developed jointly by the University of Oxford and AstraZeneca, hinges on a chimpanzee adenovirus vaccine vector (ChAdOx1) fused with the SARS-CoV-2 spike protein (Watanabe et al., 2021[85]). Promising results from animal studies indicate a reduction in disease severity upon exposure to the virus. Similarly, CanSino Biological Inc. and Beijing Institute of Biotechnology's adenoviral vector vaccine (Ad5-nCoV) is undergoing Phase I/II trials (Barajas-Nava, 2021[9]). While novel DNA and mRNA technologies hold promise, they remain unproven in human applications, with Moderna's existing mRNA-based vaccine programs still in Phase II. Recent data demonstrates that Moderna's mRNA-1273 prompts protective antibody titers in early-phase trials, accelerating progress towards Phase II (Bakhiet and Taurin, 2021[7]). Contrastingly, established protein-based vaccine platforms, including recombinant-protein, viral-vector, attenuated, or inactivated vaccines, have already demonstrated efficacy in various infectious diseases. In the fight against SARS-CoV-2, multiple approaches are being investigated, exemplified by China National Pharmaceutical Group's inactivated vaccine trials and the efforts of Sinopharm, Sinovac Biotech, and others.

Live-attenuated vaccines bear inherent immunogenic properties however it is crucial to conduct thorough safety assessments to prevent potential reversion to pathogenic forms. Robust research has been conducted on SARS-CoV live-attenuated vaccines, demonstrating their stability. Similarly, recombinant-protein and inactivated vaccines, though safer, might necessitate adjuvants for enhanced immunogenicity. The role of adjuvants, particularly in the context of SARS-CoV-2, is dual: they could bolster vaccine efficacy, especially in high-risk individuals, and optimize vaccine production scalability (Dheyab et al., 2021[17]). Advancements in adjuvant technologies include MF59, introduced in influenza vaccines for the elderly, and others like AS03, AF 03, AS 01, and AS04, each designed to enhance the immune response. These adjuvants could play a pivotal role in augmenting vaccine efficiency and streamlining production timelines, amplifying the arsenal against SARS-CoV-2.

### 5.2 Gene therapy

Nanoparticles are currently under scrutiny for their potential in delivering gene therapies aimed at addressing genetic respiratory conditions like cystic fibrosis (Ruigrok et al., 2021[63]). The intricate biopolymeric structure of respiratory mucus can pose challenges for the dispersion of nanocomplexes, either through steric hindrance or interactions with the nanocomplexes themselves. Non-cross-linked macromolecules may adhere to nanocomplex surfaces, potentially leading to aggregation and hindrance of their mucus traversal. Interestingly, studies indicate that even in the absence of aggregation, the binding of extracellular components to nanocomplex surfaces can markedly diminish their gene transfection efficacy. Consequently, the journey of nanocomplexes towards epithelial cell surfaces hinges on several variables, including aspects such as their movement within mucus, the thickness of the mucus layer, and the rate at which mucus is cleared. When nanocomplexes interact with constituents found in mucus, a series of adverse effects can occur. These effects include their entrapment within the mucus matrix, neutralization of their surface charges leading to aggregation, release of their DNA cargo, and a reduction in cellular uptake. This decreased cellular uptake results from the masking of their positive charges or receptor ligands. In essence, the complex interaction between nanocomplexes and the intricate environment of respiratory mucus significantly impacts their movement, functionality, and potential for successful gene transfection.

Numerous research studies have delved into the influence of sputum, particularly from individuals with cystic fibrosis (CF), on the structure and transfection efficiency of nanocomplexes. In one study, lipoplexes composed of 1,2-Dioleoyloxy-3-(trimethylammonium) propane (DOTAP)/DOPE were exposed to varying amounts of mucin, linear DNA, or albumin (Di Gioia et al., 2015[18]). The interaction between charged mucus components and cationic nanocomplexes resulted in a decrease or reversal of the nanocomplexes' surface charge. Increased levels of albumin and linear DNA created an anionic shield around lipoplexes, enhancing protection against aggregation. Mucin-coated lipoplexes, while avoiding substantial aggregation or dissociation, exhibited reduced transfection efficacy. This observation suggests that mucins may influence the intracellular trafficking of complexes during transfection.

The albumin-coated PEI polyplexes exhibited superior gene transfer efficiency compared to unmodified PEI, even in the presence of CF sputum (Di Gioia et al., 2015[18]) (Aguilar et al., 2020[2]). The presence of endogenous DNA within mucus is another crucial factor contributing to the decreased transfection efficiency of nanocomplexes. Experiments conducted by Alton and colleagues using cell cultures covered with diluted CF sputum revealed that gene delivery, mediated by both lipoplexes and adenoviral vectors, experienced a reduction due to CF sputum DNA binding to the epithelial cell surface (Dheyab et al., 2021[17]). In fact, a significant decline in gene transfer occurred in cells pre-coated with highly diluted sputum, as well as in those subjected to sputum removal before transfection. The transfection capability of both vectors was partially restored following pre-treatment of cells with recombinant human deoxyribonuclease (rhDNase). These findings underscore the intricate interplay between nanocomplexes and mucus components, shedding light on the multifaceted dynamics that shape transfection outcomes.

The development of an effective non-viral gene delivery system relies on its capacity to facilitate efficient cell delivery while minimizing interactions with extracellular components like mucus and avoiding detection by macrophages. In the domain of nanocomplex-based gene delivery, maintaining an overall positive surface charge of polyplexes is a common prerequisite to ensure stable complexes and achieve high transfection rates. But this positive surface charge presents challenges in interactions with extracellular anionic components. Particularly in gene transfer to the lungs, obstacles arise due to interactions with mucus components such as proteoglycans, glycosaminoglycans, and, to some extent, constituents of the surfactant layer like phospholipids. To tackle these challenges, various shielding strategies have emerged to counteract the self-aggregation of non-viral gene carriers and mitigate interactions with extracellular components. Among these strategies, the incorporation of hydrophilic, uncharged polymers like PEG (polyethylene glycol) stands out as a critical factor in facilitating effective pulmonary gene delivery. The inclusion of PEG results in more stable polyplexes while concurrently reducing interactions with sialic acid, a key component of mucus (Nguyen et al., 2008[50]). The concept of using PEG to modify drugs, proteins, and particles is not novel; its conjugation has been extensively explored to extend the circulation time of various agents, ranging from drugs to particulate drug carriers, following intravenous administration. In essence, these strategies represent innovative approaches to surmount the challenges posed by mucus interaction and cellular recognition, thereby advancing the potential for successful non-viral gene delivery to the lungs. Two distinct strategies have been employed to create nanocomplexes featuring inert polymers on their surface. The initial approach involves covalently linking the non-viral cationic carrier with the protective polymer and then mixing it with DNA. But in this method, the presence of shielding polymers might hinder the self-assembly process between the cationic carrier and DNA. The second strategy entails covalently attaching the shielding polymer to pre-formed non-viral nanocomplexes. Since PEGylation could potentially impede endosomal escape, several research groups are actively developing strategies wherein nanocomplexes shed their PEG chains either outside the cell or within acidifying endosomal compartments. Prominent among the successful nanocomplexes for lung gene delivery are synthetic cationic lipids like GL67. PEGylated GL67/DOPE lipoplexes exhibited uncompromised gene transfer efficiency even in the presence of certain factors (Sanders et al., 2002[64]).

### 5.3 Cancer immunotherapy

Nanoparticles are used to deliver immune checkpoint inhibitors or antigens to lung cancer cells, enhancing the immune response against tumors. ARAC, an immunotherapy based on nanoparticles, amplifies the effectiveness of PD-L1 inhibitors in advanced non-small cell lung cancer (NSCLC) (Reda et al., 2022[59]). This approach simultaneously delivers a PLK1 inhibitor (volasertib) and a PD-L1 antibody, targeting the overexpression of PLK1 in cancer. In a metastatic lung tumor model, ARAC lowers the required doses of volasertib and PD-L1 antibody by a factor of five, demonstrating efficacy in another lung tumor model as well. This study highlights the need for superior immunotherapy strategies.

Adjuvants imitate molecules resembling those produced by infectious pathogens, which are identified by pattern recognition receptors (PRRs), and act as immune response boosters. Frequently utilized adjuvants in cancer immunotherapy include compounds such as 3-O-desacyl-4'-monophosphoryl lipid A (MPLA), lipopolysaccharide (LPS), CpG oligodeoxynucleotides (CpG ODNs), polyinosinic: polycytidylic acid (poly I:C), and stimulator of IFN genes (STING) agonists (Park et al., 2018[54]). These adjuvants facilitate heightened anti-cancer immune responses when taken up by antigen-presenting cells (APCs) along with tumor antigens. As a result, various approaches have been devised to efficiently transport adjuvants into antigen-presenting cells (APCs) using nanoparticles. For example, adjuvants such as CpG ODNs, poly I:C, and STING agonists, which possess a negative charge, can establish electrostatic complexes with positively charged nanoparticles or polymers (Alvarez et al., 2021[4]). This enables their internalization by APCs through endocytosis, consequently bolstering the promotion of anti-cancer immunity. The utilization of nanoparticles for adjuvant delivery, combined with the direct delivery of antigens into the cytoplasm of APCs, significantly contributes to the stimulation of potent, antigen-specific T-cell responses. The integration of nanoparticle-mediated cancer antigen delivery with immune checkpoint blockade approaches can further enhance anti-cancer immune responses. Thus, nanoparticle-based strategies hold considerable promise for addressing various blood and solid cancers.

Regulatory T cells (Tregs) are a subset of T cells with immunosuppressive capabilities that can hinder the actions of anti-tumor T-effector cells (Gao et al., 2022[21]). While Tregs serve to prevent autoimmune diseases by promoting immune tolerance to self-antigens, their presence within the cancer context can impede immune responses against tumors by dampening anti-cancer immune activities within the tumor microenvironment (TME). To initiate a potent anti-tumor immune response, tactics can be utilized to actively restrain or eliminate Tregs. A prevalent strategy in lung cancer immunotherapy focuses on modulating Treg activity via checkpoint blockade. This is illustrated by substances designed to target molecules such as CTLA-4 (Gao et al., 2022[21]). Engineered nanoparticles hold promise in selectively targeting Tregs and facilitating their removal from the tumor microenvironment, thereby contributing to enhancing anti-cancer immune responses.

### 5.4 Asthma and allergy therapies

Nanoparticles can deliver anti-inflammatory agents to mitigate symptoms of asthma and allergic reactions in the respiratory system. Si-RNA-based therapy has potential for treating asthma and COPD by reducing toxicity and immunogenicity in airway inflammatory cells (Perl et al., 2010[55]). Kumar and colleagues showcased the potential of chitosan interferon-γ-plasmid DNA nanoparticles in mitigating allergen-induced airway inflammation and hyperresponsiveness (Kumar et al., 2003[37]). There have been advancements in the delivery of inhalable siRNA using poly (amidoamin) dendrimer carriers and dendrimer-based nanoparticles for in vivo applications.

Research is focusing on developing drug nanocarriers to deliver asthma therapeutics to inflammation sites. The anti-inflammatory effects of theophylline were enhanced through intranasal delivery using thiolated chitosan nanoparticles (Lee et al., 2006[39]). Muco-adhesive nanoparticles and chitosan-hyaluronic acid nanoparticles loaded with heparin have demonstrated potential in addressing asthma-related conditions (Ahmad, 2022[3]). Heparin-loaded chitosan-cyclodextrin nanoparticles have exhibited significant improvements in drug efficacy, making them promising candidates for asthma treatment.

Nanoparticles loaded with steroids have shown remarkable therapeutic benefits in comparison to free drug formulations. Research has indicated that betamethasone phosphate-loaded polymeric nanoparticles accumulate at sites of airway inflammation and exert anti-inflammatory effects (Howard et al., 2014[25]). Self-assembling nanoparticles containing dexamethasone specifically target the lungs, reducing allergic lung inflammation and airway hyperresponsiveness. Combining therapeutic peptides and dexamethasone (Dexa) within the same nanocarrier has been effective in reducing neutrophilic pulmonary inflammation (Kim et al., 2011[34]). Efforts are also underway to develop oral dispersible tablets containing prednisolone-loaded chitosan nanoparticles for paediatric asthma management.

### 5.5 Pulmonary hypertension treatment

Nanoparticles are being explored for targeted delivery of vasodilators to treat pulmonary hypertension. Pulmonary arterial hypertension (PAH) is a severe cardiopulmonary condition resulting from the remodeling of pulmonary arteries (Rubin, 2006[62]). Existing treatments aim to alleviate PAH symptoms, but a co-delivery system has been developed to target p53 and baicalein, showing promise in mitigating the disease (Teng et al., 2022[76]). This system effectively homes in on pulmonary arterial smooth muscle cells (PASMCs) in vitro, allowing for gene transfection, apoptosis induction, and inflammation suppression. In in vivo experiments, it successfully reverses PAH induced by monocrotaline, reducing pulmonary artery pressure, downregulating TNF-α, and inhibiting pulmonary arteries and right ventricular remodeling. The robust efficacy may be linked to the activation of the Bax/Bcl-2/Cas-3 signaling pathway.

## 6. Design and Development of Nanoparticle-Based TLR Agonists for Respiratory Immunotherapy

### 6.1 Rationale behind using nanoparticles for TLR agonist delivery

TLRs play a pivotal role in triggering immune responses by identifying patterns associated with pathogens (PAMPs) and patterns associated with danger (DAMPs). TLR agonists can activate immune cells and enhance their responses against infections, tumours, and other diseases. However, direct administration of TLR agonists can lead to systemic side effects and limited targeting to the desired site. Nanoparticles provide an attractive solution to these challenges due to their unique properties. They enable targeted delivery to immune cells in the respiratory system, ensuring efficient TLR agonist stimulation with minimal side effects. The controlled release of agonists from nanoparticles extends their presence at the site of action, preventing rapid clearance. Encapsulation shields agonists from degradation, preserving their potency during transport. Facilitating cellular uptake, nanoparticles enhance immune activation. In essence, nanoparticles provide a multi-faceted strategy to address challenges, optimizing therapeutic outcomes.

### 6.2 Selection of appropriate TLR agonists and compatibility with nanoparticles

The choice of TLR agonists and their compatibility with nanoparticle formulations is crucial for effective immunotherapy. Several TLR agonists have been investigated for respiratory applications:

**TLR4 Agonists:** Lipopolysaccharides (LPS) derived from bacteria are TLR4 agonists. Their strong pro-inflammatory responses and potential toxicity make them challenging candidates. Modified LPS or other TLR4 ligands with reduced toxicity may be considered. Lipopolysaccharide (LPS) serves as a potent immunomodulatory agent, activating TLR-4 in Gram-negative bacteria. Its clinical utility is restricted due to its associated toxicity. Monophosphoryl lipid A (MPL), derived from Salmonella minnesota R595, induces similar cytokine profiles to LPS but with reduced toxicity, making it a subject of extensive investigation in clinical applications (Michaud et al., 2013[47]). MPL vaccines have been assessed for their capacity to boost systemic and mucosal immune responses against hepatitis B surface antigens, tetanus toxoid, and influenza. Both MPL and MPL-mimetics show potential as therapeutic agents in modulating the innate immune response to provide short-term resistance against infectious challenges. Co-administering Francisella tularensis-LPS with MPL or MPL alone has been found to protect mice from F. tularensis infection (Richard et al., 2017[61]). Fimbriae H protein (FimH) acts as a ligand for TLR-4 and induces pulmonary changes in mice, thereby reducing mortality and morbidity associated with influenza infection. Synthetic mimetics of lipid A known as AGPs require TLR-4 recognition. Prophylactic treatment of mice with AGPs prior to inhalation of F. novicida resulted in reduced bacterial burden in the lung, liver, and spleen upon challenge, along with lower mortality rates compared to PBS-treated mice.

**TLR3 Agonists:** TLR-3 agonists, such as polyinosinic:polycytidylic acid (PIKA) and polyinosinic-polycytidylic acid (Poly IC:LC), have potent anti-viral actions and are associated with viral infection (Mifsud et al., 2014[48]). PIKA is a synthetic agonist that recruits macrophages, neutrophils, and plasmacytoid DCs into the lungs, reducing viral burden in mice infected with various influenza viruses. Poly IC:LC, a chemically stabilized variant of polyinosinic IC, activates TLR-3, providing defense against lethal challenges from avian influenza viruses (Mifsud et al., 2014[48]). Administering Poly IC:LC twice daily after infection enhances bacterial replication and leads to extensive lung necrosis in treated mice. The research conducted by Antonelli et al. advises caution in the use of immunomodulatory agents that stimulate Type 1 interferon (IFN) in regions where tuberculosis is prevalent (Antonelli et al., 2006[6]). In mice, intranasal delivery of two doses of Poly IC has been observed to increase the bacterial burden following infections with *S. pneumoniae* and methicillin-resistant *S. aureus*. Type 1 IFNs do not aid in bacterial clearance, and elevated levels of Type 1 IFNs render mice more vulnerable to *S. pneumoniae* infection. These interactions between viruses and bacteria are pertinent to human infections, as viral infections can predispose individuals to secondary bacterial infections, especially in cases of influenza A infections.

**TLR7/8 Agonists:** Imidazoquinoline compounds such as Resiquimod and Imiquimod are TLR7/8 agonists. They activate immune responses and have shown promise in cancer immunotherapy (Vasilakos and Tomai, 2013[79]). TLR-7, a receptor recognizing single-stranded RNA fragments within endosomes, is commonly activated during viral infections. Imiquimod, a potent TLR-7 agonist, triggers innate and adaptive immune pathways by inducing pro-inflammatory cytokines. Initially FDA-approved for genital wart treatment in 1997, Imiquimod's efficacy was demonstrated in a guinea pig model, reducing HSV-2 lesion frequency. Infectious agents like viruses and bacteria can contribute to cancer, with human papilloma viruses linked to basal cell carcinomas. Topical 5% Imiquimod cream displayed enhanced clearance rates for these conditions, highlighting immunostimulants' potential in cancer treatment (Vasilakos and Tomai, 2013[79]). Although extensively used for dermatologic ailments, Imiquimod can cause side effects, limiting its utility; improved variants could mitigate these concerns.

**TLR9 Agonists:** CpG oligonucleotides are TLR9 agonists that mimic bacterial DNA motifs. They induce robust immune activation and are being explored for various respiratory diseases. CpG, activating the innate immune system through TLR-9, mimics bacterial DNA's effects via non-methylated CpG motifs (Vollmer and Krieg, 2009[81]). Administering CpG-ODN shifts the Th2-biased response in Balb/c mice infected with Leishmania major towards a protective Th1 profile, rendering them resistant. This protection extends to intra-cellular pathogens like L. major and F. tularensis with durable effects independent of inoculation route, suggesting therapeutic potential. TLR-9's significance in countering HSV-2 infections is evident, as CpG-ODN administration limits viral replication and enhances survival without direct viral inhibition, instead promoting immune responses (Dar et al., 2009[16]). The CpG-ODN's efficacy surpasses other TLR ligands due to its induction of IFN-β, essential for protection. In a study comparing CpG-ODN and Resiquimod (R-848) for HSV-2 treatment, CpG-ODN's localized cytokine response proves superior, while R-848 triggers systemic chemokine release without virus control (Tomai et al., 2007[78]). Repetitive CpG-ODN administrations for therapeutic use raise concerns, as prolonged TLR activation may lead to organ pathology and harmful effects, necessitating a balanced approach for effective immunotherapies.

The compatibility between selected TLR agonists and nanoparticle formulations must consider physicochemical properties, such as solubility, charge, and hydrophobicity, to ensure stable encapsulation and release characteristics.

## 7. Synthesis and Characterization of Nanoparticle-TLR Agonist Complexes

The synthesis and characterization of nanoparticle-TLR agonist complexes represent a cutting-edge frontier in the field of immunotherapy and nanomedicine. These complexes hold tremendous promise for revolutionizing the way we harness the power of the immune system to combat diseases, particularly in the context of respiratory ailments. By merging the unique properties of nanoparticles with the potent immunostimulatory abilities of TLR agonists, researchers are striving to create innovative therapeutic platforms capable of precise immune modulation. This convergence of nanotechnology and immunology opens up exciting avenues for the development of novel treatments that can target respiratory diseases such as infections, allergies, and even cancer with enhanced specificity, efficiency, and safety. The synthesis and characterization of nanoparticle-TLR agonist complexes involve several steps:

**1. Nanoparticle Synthesis:** Nanoparticles can be synthesized using various methods such as nanoprecipitation, emulsion, or solvent evaporation. The choice of materials, size, and surface modifications depends on the targeted immune cells and desired release kinetics.

**2. Encapsulation of TLR Agonists:** TLR agonists can be encapsulated within nanoparticles during or after their synthesis. Techniques like co-precipitation, solvent displacement, or layer-by-layer assembly can be employed. Encapsulation of TLR agonists within nanoparticles presents a versatile approach that enhances their therapeutic efficacy and mitigates potential drawbacks (Katebi et al., 2021[31]). This strategy involves the incorporation of TLR agonist molecules into nanoparticle carriers, creating a protective environment that offers controlled release, improved stability, and targeted delivery to immune cells (Katebi et al., 2021[31]). Various encapsulation techniques have been employed, such as co-precipitation, solvent displacement, and layer-by-layer assembly, each with distinct advantages and applications.

Co-precipitation is a widely used technique where TLR agonist molecules and the materials forming the nanoparticle matrix are precipitated together from a solution. This method ensures uniform distribution of the agonists within the nanoparticles, promoting controlled release and protection from enzymatic degradation (Sharma et al., 2023[66]). 

Solvent displacement involves dissolving both the TLR agonist and the nanoparticle matrix material in a common solvent and then introducing a non-solvent, causing nanoparticle formation. This technique allows for controlled particle size and drug loading. Research encapsulated the TLR-4 agonist monophosphoryl lipid A (MPLA) using solvent displacement into lipid-based nanoparticles (Cordeiro et al., 2015[14]). These nanoparticles exhibited improved stability and controlled release of MPLA, enhancing the immune response against cancer cells.

Layer-by-layer assembly is a more complex technique where multiple layers of materials are alternately deposited on the nanoparticle's surface, forming a shell around the TLR agonist-loaded core. This method allows precise control over drug release rates and provides opportunities for functionalization. For example, a study by Jia et al. (2023[30]) used layer-by-layer assembly to encapsulate the TLR-9 agonist CpG oligodeoxynucleotides (CpG ODN) within gold nanoparticles. This approach enabled sustained release of CpG ODN and significantly improved the immune response in a tumor model.

**3. Characterization:** The nanoparticle-TLR agonist complexes need to be characterized for size, morphology, surface charge, drug loading efficiency, and drug release kinetics. Techniques such as dynamic light scattering, transmission electron microscopy, zeta potential measurement, and spectroscopy can be used. Characterizing nanoparticle-TLR agonist complexes is crucial to ensure their effectiveness, stability, and suitability for targeted immune modulation. Several key parameters, including size, morphology, surface charge, drug loading efficiency, and drug release kinetics, must be thoroughly assessed using a range of advanced techniques to gain a comprehensive understanding of their properties. Dynamic Light Scattering (DLS) is a technique commonly employed to measure particle size distribution in solution. By analyzing the intensity fluctuations of scattered light, DLS provides information about the average particle size and size distribution of the nanoparticle-TLR agonist complexes. For instance, Wang (2019[83]) used DLS to determine the particle size of polymeric nanoparticles encapsulating a TLR-3 agonist, providing insights into the formulation's stability and potential for immune activation (Wang, 2019[83]; Sui et al., 2022[72]). Transmission Electron Microscopy (TEM) offers high-resolution imaging that enables the visualization of nanoparticle morphology and structure. TEM provides valuable information about the shape, uniformity, and core-shell structure of nanoparticle-TLR agonist complexes. Research by Wang et al. (2022[82]) employed TEM to examine the morphology of gold nanoparticles loaded with a TLR-9 agonist, demonstrating successful encapsulation and confirming their structure. Zeta Potential Measurement is utilised to determine the surface charge of nanoparticle complexes. The surface charge influences particle stability and cellular interactions. Nanoparticles with appropriate surface charges can improve cellular uptake and enhance immune responses. For example, Wang et al. (2021[84]) investigated zeta potential values to optimize cationic lipid-based nanoparticles encapsulating a TLR-7 agonist, aiming to enhance antigen-presenting cell uptake and immune activation. Spectroscopic techniques, such as UV-Vis absorption and fluorescence spectroscopy, can assess drug loading efficiency and release kinetics. UV-Vis absorption can quantify the amount of loaded TLR agonist, while fluorescence spectroscopy can monitor release profiles by measuring changes in fluorescence intensity upon TLR agonist release. 

**4. In vitro and In vivo Evaluation:** The assessment of nanoparticle-TLR agonist complexes doesn't conclude with characterization; it extends to in vitro and in vivo evaluations that provide valuable insights into their immune-modulating potential and therapeutic applications. These evaluations involve a series of carefully designed experiments that collectively determine the effectiveness of the complexes in eliciting desired immune responses and their potential as therapeutic agents.

*4.1 In vitro Evaluation:* In vitro studies employ immune cell assays to examine the immune-stimulating properties of nanoparticle-TLR agonist complexes. These assays typically involve exposing immune cells, such as dendritic cells, macrophages, or lymphocytes, to the complexes and monitoring their responses. Key parameters evaluated include cytokine secretion (e.g., interferons, interleukins), expression of co-stimulatory molecules, and cellular activation markers. These assays provide valuable insights into the ability of the complexes to trigger immune activation and modulation. For instance, researchers may assess the ability of TLR agonist-loaded nanoparticles to enhance dendritic cell maturation and antigen presentation, which are essential steps in initiating adaptive immune responses.

*4.2 In vivo Evaluation:* In vivo studies are essential to understand how nanoparticle-TLR agonist complexes perform in a more complex biological context. These studies are typically conducted using relevant animal models, such as mice, to assess immune responses, targeting efficiency, and therapeutic efficacy (Wang et al., 2021[84]; Haegebaert et al., 2022[22]). In these studies, animals are administered the nanoparticle complexes, and various parameters are measured:

- Immune Responses: Researchers evaluate immune responses by measuring cytokine levels, antibody production, and cellular immune activation. This provides insights into the extent and nature of the immune activation triggered by the complexes.
- Targeting Efficiency: The ability of the complexes to selectively target immune cells or specific tissues is assessed. This involves tracking the distribution of fluorescently labeled or radiolabeled complexes in the body and analyzing their accumulation in target tissues.
- Therapeutic Efficacy: In disease models, the complexes' therapeutic potential is evaluated by monitoring disease progression, survival rates, and tissue pathology. For example, the impact of TLR agonist-loaded nanoparticles on tumor growth inhibition or infection clearance can be assessed.
- Safety Profile: In vivo studies also examine potential adverse effects, such as systemic inflammation, tissue damage, or off-target immune activation. These evaluations are crucial for determining the safety of the nanoparticle complexes.

By integrating in vitro and in vivo evaluations, researchers gain a comprehensive understanding of the immune-modulating capabilities and therapeutic potential of nanoparticle-TLR agonist complexes. These evaluations guide the optimization of complex formulations, dosing regimens, and delivery strategies, ultimately contributing to the development of effective and safe immunotherapies. The findings from these evaluations inform subsequent preclinical and clinical studies, facilitating the translation of these strategies to clinical applications.

**- Safety Considerations:** Safety considerations are paramount in the development of nanoparticle-TLR agonist complexes as potential immunotherapies. Comprehensive evaluations are essential to ensure that these complexes do not lead to unintended immune activation, cytotoxicity, or other adverse effects. A systematic approach involving in vitro, in vivo, and mechanistic studies is adopted to assess safety profiles rigorously. In cell-based assays, the potential cytotoxicity of nanoparticle-TLR agonist complexes is assessed by exposing various cell lines to increasing concentrations of the complexes. Cell viability assays, such as MTT or CellTiter-Glo, are employed to determine if the complexes induce cell death or compromise cell viability (Teow et al., 2019[77]). The potential for excessive immune activation is evaluated by monitoring the secretion of pro-inflammatory cytokines upon exposure to the complexes. Shi and Gu in 2023 investigated the cytotoxicity and immune response induced by TLR-7 agonist-loaded nanoparticles in dendritic cells, ensuring their safety for subsequent in vivo studies (Shi and Gu, 2023[69]). Animal studies are crucial for assessing the safety of nanoparticle-TLR agonist complexes in a physiological context. Researchers evaluate whether administration of the complexes leads to systemic inflammation, organ damage, or other adverse effects. Detailed histopathological analysis of various organs is conducted to identify any signs of toxicity or abnormal tissue morphology. Blood chemistry and haematology analyses provide insights into potential systemic disturbances. For example, Marasini et al. (2017[44]) studied the in vivo safety of CpG-loaded nanoparticles in mice and concluded that the administration did not induce significant systemic toxicity (Marasini et al., 2017[44]). Understanding the mechanisms underlying potential adverse effects is crucial. Mechanistic studies delve into how nanoparticle-TLR agonist complexes interact with immune cells, their uptake mechanisms, and their impact on immune signaling pathways. This knowledge aids in pinpointing any potential triggers of excessive inflammation or cytotoxicity. The insights gained from safety evaluations in preclinical studies guide the design of clinical trials. The translation of nanoparticle-TLR agonist complexes into human therapies necessitates rigorous safety assessments in controlled clinical settings. Safety parameters such as cytokine levels, immune activation markers, and potential adverse events are monitored meticulously during these trials.

## 8. Preclinical Studies and Efficacy

**Figure 4:**
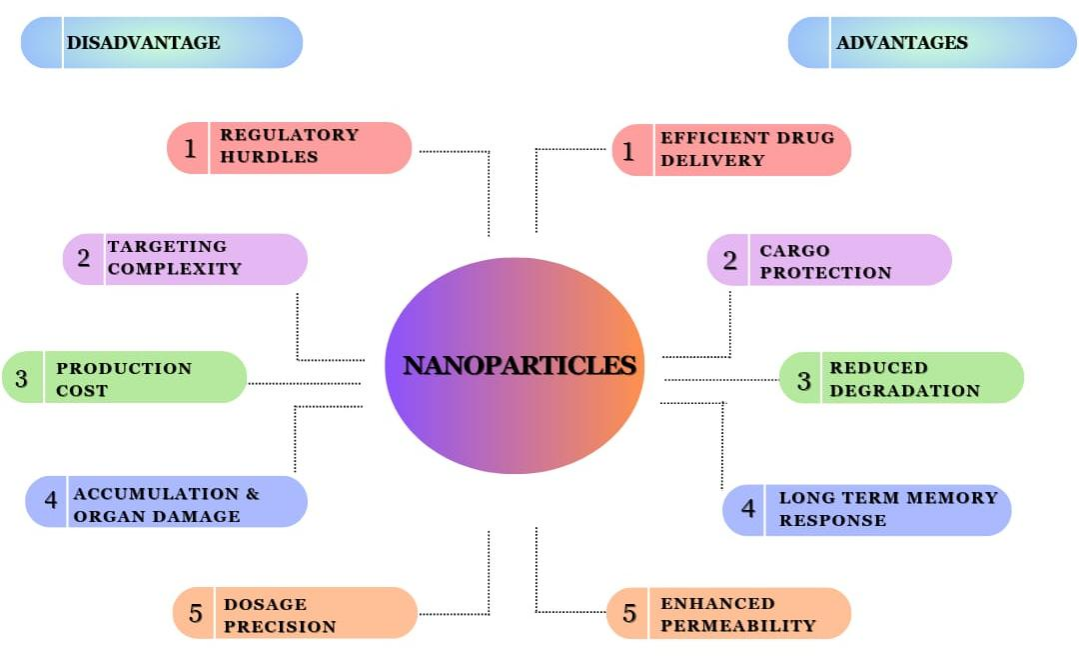
Advantages and limitations of nanoparticle-based toll-like receptor agonists for respiratory immunotherapy. In the pursuit of innovative therapies for respiratory diseases, preclinical studies involving animal models have played a pivotal role in evaluating the potential effectiveness and safety of various TLR agonists. Toll-like receptors are integral components of the immune system, and harnessing their activation has emerged as a promising avenue for combating respiratory ailments. The following overview encapsulates the outcomes of diverse preclinical investigations, shedding light on the impact of TLR-targeted interventions on respiratory conditions. From the modulation of immune responses to the intricate study designs employed, these trials collectively contribute to the evolving landscape of respiratory immunotherapy.

Nanoparticle-Based TLR Agonists hold promise as a novel approach to respiratory immunotherapy, offering both advantages and disadvantages as shown in Figure 4(Fig. 4). On the positive side, these nanoparticles can enhance the precision and efficiency of immune responses within the respiratory system. By mimicking the natural signaling pathways of the immune system, they can stimulate a targeted and potent immune response against respiratory pathogens and allergens. Nanoparticles can be designed for sustained release, prolonging the therapeutic effect and reducing the need for frequent dosing. There are also challenges associated with this technology. First, concerns regarding nanoparticle toxicity and safety must be addressed thoroughly. The precise design and formulation of nanoparticles can be complex, potentially leading to high production costs and regulatory hurdles. Individual variability in immune responses may pose a challenge in achieving consistent therapeutic outcomes. Table 1(Tab. 1) outlines selected TLR-targeted agents and corresponding study designs evaluated in respiratory disease models.

## 9. Impact of Nanoparticles on the Immune System

Nanoparticles (NPs) have shown significant potential as drug delivery vehicles, vaccine adjuvants, and immunomodulatory agents due to their small size, large surface area, and tunable physicochemical properties. Their interaction with the immune system can lead to either immunostimulation or immunosuppression, depending on the type of nanoparticles, their surface modifications, and the biological environment they encounter. Understanding these interactions is critical for determining whether NPs can enhance or suppress immune responses and whether they carry a risk of causing a cytokine storm (Table 2(Tab. 2)).

### Immunostimulatory effects of nanoparticles

Some nanoparticles have been designed to enhance immune responses, making them valuable in cancer immunotherapy, vaccine adjuvants, and for treating infectious diseases. Nanoparticles can be engineered to increase the production of pro-inflammatory cytokines (e.g., TNF-α, IL-6, IL-1β, and IFN-γ) and enhance antigen presentation. They can also activate Toll-like receptors (TLRs), particularly TLR4, and stimulate the function of immune cells like dendritic cells (DCs), macrophages, and T cells. However, overstimulation of these pathways can increase the risk of cytokine storms, a severe inflammatory response that can lead to tissue damage and systemic toxicity.

### Immunosuppressive effects of nanoparticles

Conversely, some nanoparticles can exert immunosuppressive effects, making them useful in eating autoimmune diseases or in reducing inflammation in chronic conditions. These nanoparticles can reduce the release of pro-inflammatory cytokines and promote the secretion of anti-inflammatory cytokines (e.g., IL-10, TGF-β). They may also induce regulatory T cells (Tregs) and impair the maturation of dendritic cells, which dampens the overall immune response. Nanoparticles can also inhibit the function of certain immune cells, including macrophages and neutrophils, which helps in preventing excessive immune reactions.

### Potential for cytokine storm induction

Nanoparticles that strongly stimulate immune responses pose a risk for inducing a cytokine storm, particularly if they activate pathways involving TLRs and pro-inflammatory cytokine production. This phenomenon has been observed in some viral infections and immunotherapies and can lead to uncontrolled inflammation. Nanoparticles that activate TLR4, in particular, have been shown to induce a cascade of cytokine release, potentially leading to a cytokine storm. Proper formulation and surface modification are crucial to mitigate this risk while maintaining the desired therapeutic effects.

## 10. Safety and Toxicity Considerations of Nanoparticle-Based Therapies

The advancement of nanoparticle-based therapies, including those employing TLR agonists, mandates a rigorous assessment of safety to ensure their clinical feasibility. Numerous concerns necessitate thorough investigation to ascertain the viability and potential risks of these therapeutic approaches:

*Off-target effects:* Nanoparticles, due to their small size and unique properties, have the potential to accumulate inadvertently in non-target tissues. This accumulation could result in unintended immune activation, inflammation, or adverse reactions. A study by Huang et al. (2022[26]) highlighted concerns over non-specific accumulation of TLR agonist-loaded nanoparticles in the liver, underscoring the need for precise targeting strategies to mitigate off-target effects (Huang et al., 2022[26]).

*Immunogenicity:* Nanoparticles themselves might trigger immune responses due to their foreign nature, leading to potential immunotoxicity or undesirable immune reactivity. Researchers must assess if the nanoparticles induce unwanted immune activation, which could compromise their therapeutic potential. A study by Ilinskaya and Dobrovolskaia (2016[27]) demonstrated that certain nanoparticles can induce immune cell activation, emphasizing the importance of understanding their immunogenicity (Ilinskaya and Dobrovolskaia, 2016[27]).

*Organ accumulation and clearance:* Inadequate clearance of nanoparticles from the body can result in their accumulation in organs, potentially causing long-term toxicity. Researchers must evaluate the biodistribution and clearance kinetics of nanoparticle-TLR agonist complexes to prevent adverse effects associated with organ accumulation. 

*Biocompatibility:* The choice of materials for nanoparticle formulations is critical to ensure biocompatibility and avoid triggering inflammation, tissue damage, or immune reactions. Researchers must carefully select materials that are well-tolerated by the body to prevent adverse responses. 

*Dose-dependent effects:* Dosing regimens must be precisely determined to prevent excessive immune activation, which could lead to adverse effects or immune tolerance. The fine balance between therapeutic efficacy and potential toxicity must be established to optimize dosing strategies. Research by (Lutz et al., 2023[43]) highlighted the importance of dose-dependent effects of TLR agonist-loaded nanoparticles in modulating immune responses effectively.

**Figure 5:**
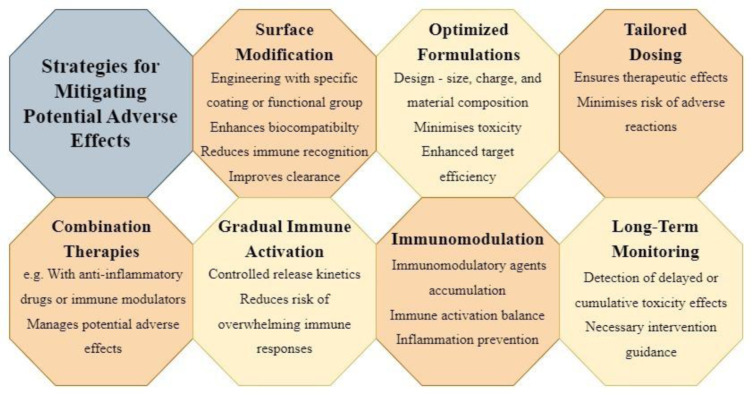
Strategies to mitigate potential adverse effects associated with nanoparticle-based therapies

Evaluating the safety of nanoparticle-based therapies involves a comprehensive approach combining in vivo toxicity studies and in vitro assessments. Figure 5(Fig. 5) depicts strategies to mitigate potential adverse effects associated with nanoparticle-based therapies. In-vivo toxicity studies using animal models are crucial to assess systemic toxicity, potential adverse effects, and organ accumulation of nanoparticle-TLR agonist complexes. Histological analysis reveals tissue damage, inflammation, or cellular alterations caused by these complexes, while blood chemistry analysis detects changes in organ function. Immune response monitoring involves evaluating cytokine levels and immune cell activation, collectively shedding light on the impact of nanoparticles on the whole organism. These studies strictly adhere to established ethical guidelines and animal welfare regulations. In vitro cellular studies expose various cell lines to nanoparticle-TLR agonist complexes to gauge cytotoxicity, effects on cell viability, and potential immune activation. Assessing both target and non-target cell lines helps uncover potential off-target effects. Standardized assays like MTT or CellTiter-Glo measure cell viability, while ELISA or flow cytometry detect cytokine release and immune activation markers, ensuring consistent evaluation across different cell types. Immunogenicity testing involves evaluating immune responses triggered by the complexes, encompassing measurements of antibody production and cytokine release. These insights discern whether the complexes induce immune reactions that might compromise their therapeutic potential, and these tests adhere to established guidelines and validated methods. For therapies targeting specific organs, such as respiratory treatments for lung diseases, organ-specific assessments are pivotal. Using lung tissue models simulates nanoparticle distribution, cellular interactions, and potential inflammatory responses within the lung, providing insights into how nanoparticle-TLR agonist complexes interact with lung tissues and whether they induce any adverse effects.

## 11. Challenges and Future Perspectives

Nanoparticle-based immunotherapy represents a cutting-edge approach with immense potential for revolutionizing the treatment landscape of respiratory diseases. This area of drug delivery faces many challenges, one of the key hurdles lies in striking a delicate balance between immune activation and potential toxicity. The intricate interplay between nanoparticle properties, TLR agonist selection, and immune response modulation demands comprehensive preclinical studies to unravel the complex mechanisms underlying their interactions. the translation from successful animal models to safe and effective human therapies remains a significant challenge, necessitating a thorough understanding of species-specific differences and the intricacies of human immune responses. The design and development of nanoparticles tailored for respiratory system targeting are inherently intricate, requiring meticulous consideration of particle size, surface properties, and formulation stability. Achieving efficient delivery to deep lung tissues while evading mucociliary clearance presents a technical challenge. The potential for immunogenicity and off-target effects requires careful scrutiny and the development of strategies to enhance biocompatibility and minimize unintended immune responses. As nanoparticle-based TLR agonist therapies advance, standardizing methods for synthesizing, characterizing, and assessing these complex formulations becomes essential. Rigorous protocols for toxicity evaluation, both in vitro and in vivo, are vital to ensure patient safety. Moreover, addressing long-term effects, such as immune tolerance or cumulative toxicity, necessitates extended monitoring and ongoing research.

Looking ahead, the future of nanoparticle-based immunotherapy remains promising. Overcoming challenges will likely involve interdisciplinary collaboration among immunologists, nanotechnologists, clinicians, and regulatory bodies. Refining nanoparticle design, harnessing personalized medicine approaches, and fine-tuning dosing regimens could unlock their full potential. Further innovation in materials science, nanotechnology, and immunology will contribute to optimizing nanoparticle-based therapies, making them a cornerstone of precision medicine in respiratory diseases. While obstacles persist, the convergence of scientific expertise and technological advancements paints a hopeful picture, where nanoparticle-based immunotherapy might significantly enhance patient outcomes and reshape the landscape of respiratory disease treatment.

## 12. Conclusion

In conclusion, the integration of nanoparticles and TLR agonists holds immense promise in reshaping the landscape of respiratory immunotherapy. The innovative potential of this approach lies in its ability to finely tune immune responses, enhance targeting efficiency, and offer a personalized treatment paradigm. As demonstrated through preclinical studies, nanoparticle-based TLR agonists showcase remarkable efficacy in ameliorating various respiratory conditions, from asthma to cancer. Several challenges, like a delicate balance between therapeutic benefits and potential adverse effects demands meticulous exploration, thorough toxicity assessments, and a deep understanding of immune modulation dynamics. The multidisciplinary collaboration of scientists, clinicians, and regulatory bodies is pivotal to navigate these challenges and pave the way for safe and effective clinical translation. The precise tuning of nanoparticle properties, rational design of formulations, and advancements in delivery techniques could propel this approach to the forefront of modern medicine. By harnessing the power of nanotechnology to modulate immune responses at the respiratory site, stands the brink of a new era where tailored therapies offer the prospect of improved outcomes, minimized side effects, and a personalized approach to patient care. As research advances and technology evolves, nanoparticle-based TLR agonist therapies may hold the key to addressing unmet needs in respiratory diseases, leading us towards a future where precision and innovation converge for the betterment of human health.

## Notes

Keshav Raj Paudel and Kamal Dua (NICM Health Research Institute and School of Science, Western Sydney University, Westmead, NSW, 2145, Australia; E-mail: k.dua@westernsydney.edu.au) contributed equally as corresponding author.

## Declaration

### Acknowledgement

Authors would like to acknowledge the APC support of through UTS institutional agreement with journal through CAUL.

### Funding

The authors declare that no funds, grants, or other support were received during the preparation of this manuscript.

### Authors contributions

Conceptualization; ASR, KD, KPR. Data curation; ASR. Formal analysis; ASR, KD. Investigation; ASR, KD. Methodology; ASR, KD. Project administration; ASR. Resources; ASR. Supervision; KD, KRP. Validation; KD. Visualization; ASR. KD. Roles/Writing - original draft: ASR, AV, SP, MBN and Writing - review & editing: KRP, ASR, AV, SP, MBN, KD.

### Declaration of interest

None.

### Artificial Intelligence (AI) - assisted technology

No artificial intelligence tools were used in the preparation, writing, editing, or analysis of this manuscript.

## Figures and Tables

**Table 1:**
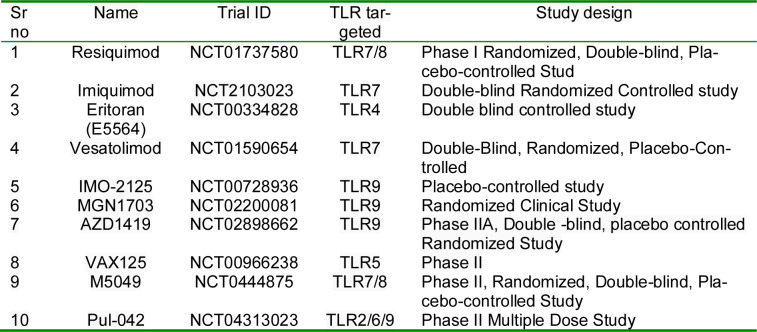
The results of preclinical studies using animal models of respiratory diseases

**Table 2:**
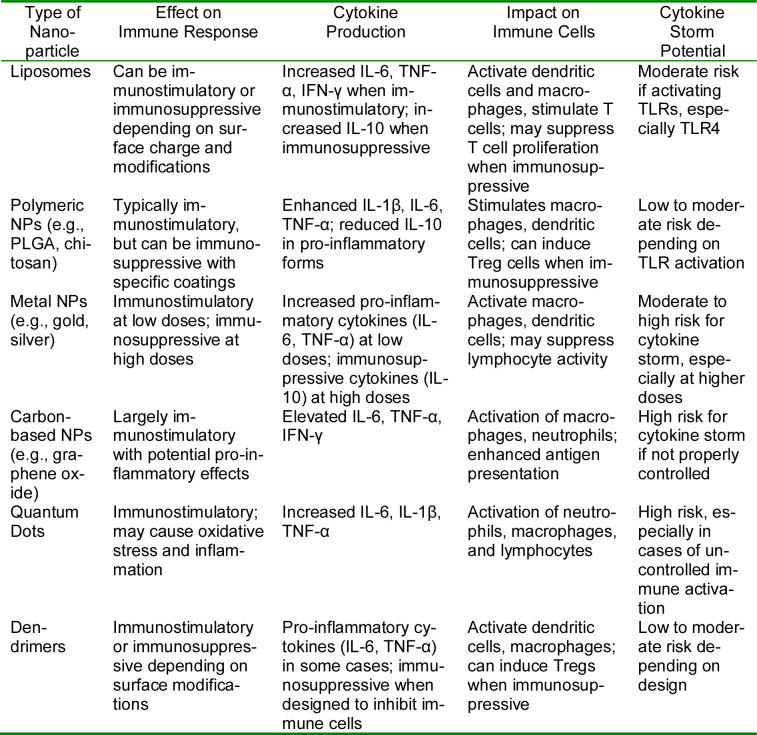
Impact of different types of nanoparticles on the immune system

**Figure 1:**
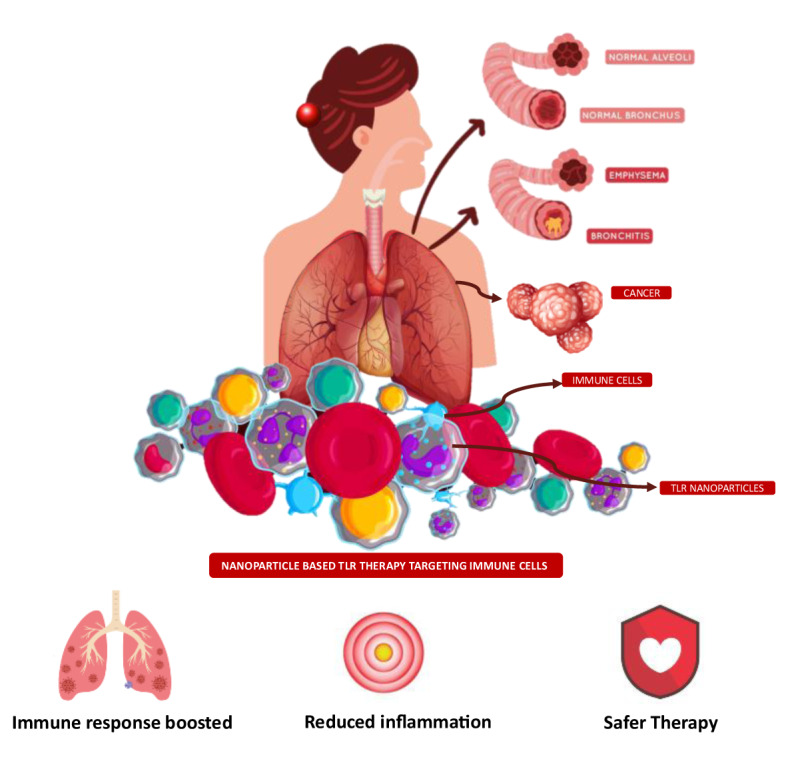
Graphical abstract
